# Treatment of patients with tumor/treatment-related hypothalamic obesity in the first two years following surgical treatment or radiotherapy

**DOI:** 10.1038/s41598-025-85262-1

**Published:** 2025-01-16

**Authors:** Hermann L. Müller, Julian Witte, Bastian Surmann, Manuel Batram, Kylie Braegelmann, Mathias Flume, Julia Beckhaus, Nicolas Touchot, Carsten Friedrich

**Affiliations:** 1 Department of Pediatrics and Pediatric Hematology/Oncology, University Children’s Hospital, Carl Von Ossietzky Universität, Klinikum Oldenburg AöR, Rahel-Straus-Straße 10, 26133 Oldenburg, Germany; 2 Vandage GmbH, Detmolder Straße 30, 33604 Bielefeld, Germany; 3 Gene Access GmbH, Seeweg 26, 44263 Dortmund, Germany; 4 Rhythm Pharmaceuticals, 222 Berkeley Street, Boston, MA 02116 USA

**Keywords:** Hypothalamic obesity, Tumor/treatment related HO, Rare sellar/suprasellar tumors, Craniopharyngioma, Endocrinology

## Abstract

Survivors of sellar/suprasellar tumors involving hypothalamic structures face a risk of impaired quality of life, including tumor- and/or treatment-related hypothalamic obesity (TTR-HO) defined as abnormal weight gain resulting in severe persistent obesity due to physical, tumor- and/or treatment related damage of the hypothalamus. We analyze German claims data to better understand treatment pathways for patients living TTR-HO during the two years following the index surgical treatment. A database algorithm identified patients with TTR-HO in a representative German payer claims database between 2010 and 2021 (n = 5.42 million patients). Claims from 37 patients with TTR-HO were analyzed on a quarterly basis over 2 years. The analysis considered inpatient, outpatient, and prescription data. In the follow-up period, patients with TTR-HO are hospitalized 3.68 times on average; 37% of hospitalizations in year 1 and 31% in year 2 are due to TTR-HO. On average, patients see a general practitioner 12.27 times and various specialists 20.45 times. The need for complex neuroendocrine therapy develops quickly, with most patients having 2–3 neuroendocrine prescriptions in any given quarter. The management of patients with TTR-HO requires frequent inpatient and outpatient visits for tumor follow-up and management of incident comorbidities, and most patients with TTR-HO require intense polytherapy.

**Supplementary Information:**

The online version contains supplementary material available at 10.1038/s41598-025-85262-1.

## Introduction

Acquired hypothalamic obesity (HO) is a rare subtype of obesity caused by acquired, non-genetic hypothalamic dysfunction^1^. In contrast to general obesity, which is linked to an increased risk of serious health conditions over time^2,3^, acquired HO is characterized by rapid weight gain due to hyperphagia and reduced metabolic rate, resulting quickly in morbid obesity, impaired quality of life, and increased mortality and morbidities^4,5^. Although acquired HO is best known in the context of craniopharyngioma, it can also occur in cases of sellar/suprasellar tumors or following irradiation, trauma, inflammation, or surgical insult to the hypothalamus. Space-occupying lesions, such as craniopharyngiomas and pituitary macroadenomas with sellar/suprasellar extension and invasion of hypothalamic nuclei^6,7^, as well as the subsequent surgical and/or radiooncological treatment^8,9^, are the most common causes of hypothalamic damage. Survivors of hypothalamic and sellar/suprasellar tumors are at high risk for significant morbidity, impacting health and health-related quality of life^10–12^. We refer to the development of HO following tumor/treatment as TTR-HO. Although it is challenging to isolate the burden of TTR-HO from the general medical burden following tumor and treatment, TTR-HO is a distinct indication with unique challenges for those who experience rapid and persistent weight gain resulting from hypothalamic damage.

TTR-HO is a rare disease and research on patient pathways is limited. Rapid weight gain can begin prior to treatment, but usually starts during the first months after surgery or radiation, leading quickly to morbid obesity and related morbidities, including cardiovascular disease, type 2 diabetes mellitus, disturbances of circadian rhythm, sleep-disordered breathing, metabolic-associated liver disease, and reduction in functional capacity^4,5,13^. Cardiovascular risk factors are prevalent in adult and pediatric patients^14,15^. Aggressive treatment of these factors is essential to reduce mortality and morbidity in patients living with HO^4,16^.

Despite several treatment options, such as medication and lifestyle modifications, clinical management of HO remains challenging^5,17^. Data on treatment pathways for patients with TTR-HO is limited^18^ and comes from non-representative sources, such as case reports or a self-reported craniopharyngioma registry^17,19^. However, robust patient characterization and improved understanding of treatment pathways is necessary to improve treatment strategies. Therefore, this study aims to provide the first characterization of treatment pathways by comprehensively analyzing resource utilization, follow-up diagnoses, and prescription medications for patients with TTR-HO.

## Methods

### Study design and database

This retrospective study was conducted according to subject privacy requirements and the Declaration of Helsinki of 1964. Patient data were completely anonymized by the data provider before being made available for research. Analyses were based on anonymized routinely collected claims data from German Statutory Health Insurance (SHI), a system which insures 88% of the German population^20^. GWQ ServicePlus AG, a data service comprised of 19 midsized SHI funds, provided the data. The dataset includes 5.42 million people, representing 6.3% of the total German SHI population. Based on a comparison with official statistics published by the German Health Ministry on SHI (“KM6”), the dataset is representative of the German SHI population in terms of age and gender distribution^21^. Analyses were based on data covering the period 01/2010 to 12/2021. The study used the years 2011–2019 for patient identification, allowing for a 1-year wash-in and 2-year post-observational period.

Diagnostic data included all diagnoses documented during inpatient stays and outpatient physician contacts. Laboratory and clinical parameters are not available in German claims data; therefore, we are not able to consider tumor size or provide results by treatment types. Inpatient medications could not be analyzed in detail, because these are not billed individually in German claims data. For this analysis, high-priced drugs with an individual inpatient expensing fee were not included. A general description of the claims database in the German setting is available from Swart et al*.*^22^.

### Study population

The dataset contains all persons in the SHI database between 0 and 100 years of age in the years 2010–2021. The analysis identified all patients hospitalized with an incident primary- or secondary-discharge tumor diagnosis known to potentially lead to TTR-HO (“index hospitalization”) and incident inpatient brain surgery/radiotherapy. Patients with TTR-HO were then validated based on incident obesity (ICD-10-GM E66.x, E67.x, E68, R63.2) within twelve months after index hospitalization, development of central diabetes insipidus (arginine vasopressin deficiency, hereafter AVP-D; ICD-10-GM E23.0, E23.2, E23.3, E23.6, E23.7) and a desmopressin prescription (ATC-code H01BA02) within twelve months after index hospitalization. AVP-D is used as a validation criterion because it is correlated with hypothalamic damage and postsurgical weight gain; although AVP-D does not cause HO, it is an effective marker of hypothalamic damage^23,24^. The study included patients with 36 consecutive months of observational period (one year before index hospitalization of tumor-related surgery/radiotherapy for incidence validation, one year after index hospitalization for HO validation, and an additional year for observation of patient pathways). The first patient entered the study in 2011 and the last patient entered in 2019. More details on TTR-HO patient selection have been published previously^25^.

### Patient pathway evaluation

We evaluated clinical resources at an individual patient level and aggregated across all observations. Resources included hospitalizations, ICU admission, follow-up after radiotherapy, total outpatient physician visits, visits per specialist group, documented primary inpatient and outpatient diagnoses, and outpatient prescription medications. We critically reviewed the claims data to distinguish between service utilization related to the index tumor/TTR-HO and those related to other medical conditions. Whenever possible, we used unambiguous ICD-10 or ATC codes to directly classify utilization as TTR-HO-related or non-TTR-HO related. For example, unambiguous tumor- and endocrinology-related diagnoses could be directly attributed to the index event. However, when ICD-10 or ATC codes could be related to several indications, a data review by clinical experts allowed for indirect classification of utilization. As the study does not include a comparison group, patient pathways described in this study are those of patients who develop TTR-HO; therefore, we cannot conclusively differentiate between medical burden resulting exclusively from other tumor sequelae and medical burden due exclusively to TTR-HO.

Use of clinical resources was recorded for each interaction or prescription on a daily basis; we aggregated these data at the quarterly and annual levels following index hospitalization. Furthermore, we examined hormone replacement therapies in detail, as these are among the most common therapies for patients with TTR-HO. These included desmopressin (ATC H01BA02), hydrocortisone (ATC H02AB09), testosterone (ATC G03BA03), somatropin (ATC H01AC01), levothyroxine (ATC H03AA01), and female sex steroids such as estrogens (ATC G03C), progestogens (ATC G03D), and their combinations (ATC G03F).

### Outcome measures and statistical analysis

We evaluated the most common diagnoses and prescriptions for identified patients in the two years after the index hospitalization. For each diagnosis, we reported the share of patients as well as the number of average interactions per patient. For prescriptions, we calculated the share of patients receiving the most frequent prescription medicines and assessed the volume of prescribed Defined Daily Doses (DDD) for each therapy. DDDs represent the assumed average maintenance dose per day for a drug used for its main indication for adults. We also assessed the number of TTR-HO patients with specific therapies and therapy combinations indicated for the treatment of hypothalamic syndrome. For each outcome measure, analysis over the 2-year period was also done by age group: < 20, 20 to 59 and 60 + years. Mean values and percentages are reported on a quarterly and annual basis. STROBE criteria were followed in reporting study results. Analyses were performed with R (Version 4.1.3), a standard and open-access statistical-computing programming language.

## Results

### Study population

Based on a dataset of 5.42 million persons over nine years, the analysis identified 3,976 patients with index hospitalizations potentially leading to TTR-HO. Of these, the analysis identified 37 patients with TTR-HO. The patients presented at a mean age of 38 years at index hospitalization (standard deviation [SD]: 18.0 years) and the majority of patients were women (59.5%).

### Inpatient visits

During the 8 quarters following the index event, the 37 patients with TTR-HO were hospitalized 3.68 times on average, 2.54 times during the first year and 1.13 times during the second year (Table 1). Of all hospitalizations, 37% in the first year and 31% in the second year had a primary diagnosis directly related to TTR-HO. Of the primary diagnoses related to TTR-HO, tumor-related follow-up visits were most frequent (88%), followed by AVP-D (9%), and hypopituitarism (3%). The primary diagnoses for non-TTR-HO-related hospitalizations varied. The most common was “encephalitis, myelitis and encephalomyelitis, unspecified” (9%). Across all types of hospitalizations, the most common secondary diagnoses were TTR-HO-related, including AVP-D (35%), hypopituitarism (21%), hypokalemia (18%), and hypothyroidism (12%, See Supplementary Table S1). In the first year, 23% of TTR-HO-related hospitalizations included an admission to ICU; this decreases to 0% in the second year. Patients younger than 20 years of age had a higher frequency of hospitalization in both years, with a total of 4.66 hospitalizations per patient over the follow-up period (See Supplementary Table S2).

**Table 1:** Frequency of HO- and non-HO-related hospitalization among patients with TTR-HO (n = 37).

Hospitalization	Outcome	Post observational period									
		Q1	Q2	Q3	Q4	Q5	Q6	Q7	Q8	Y1	Y2
All hospitalizations											
Total	Mean number per patient with TTR-HO	0.92	0.73	0.54	0.36	0.30	0.36	0.19	0.30	2.54	1.13
Hospitalizations related to the index hospitalization											
Total	Mean number per patient with TTR-HO	0.30	0.27	0.24	0.14	0.14	0.14	0.05	0.03	0.95	0.35
Total	Percent of all hospitalizations, %	32%	37%	45%	38%	45%	38%	29%	9%	37%	31%
ICU	Percent of all hospitalizations, %	18%	20%	22%	40%	0%	0%	0%	0%	23%	0%

ICU: Intensive care unit; Q: Quarter; TTR-HO: Tumor/treatment-related hypothalamic obesity; Y: Year.

### Outpatient physician vists

Patients with TTR-HO consulted a variety of outpatient physicians in the two years following the index event (Table 2). During the follow-up period, on average, patients saw a general practitioner 12.27 times and specialists 20.45 times. The most common specialists were endocrinologists/diabetologists, ophthalmologists, gynecologists, neurosurgeons, neurologists, and radiologists. In addition to visits for oncological follow-up, most patients had visits for neuroendocrine disorders, including 84% for hypopituitarism and 81% for AVP-D (Table 3). Management of hypopituitarism began early, with 27% of patients consulting for hypopituitarism in the first quarter after the index event. This rate steadily increased to 54% of patients in the fourth follow-up quarter. During the two-year follow-up, 100% of patients under 20 years of age consulted for hypopituitarism, compared to 85% of patients 20 to 60 years of age and only 60% of patients over 60 years of age (See Supplementary Table S3-S5).

**Table 2:** Outpatient contacts (mean per patient) with specialist groups within the two years following TTR-HO-associated index hospitalization (n = 37).

Specialist group	Post-observational period										
	Q1	Q2	Q3	Q4	Q5	Q6	Q7	Q8	Y1	Y2	Total
General Practitioner	1.59	1.84	1.62	1.54	1.54	1.22	1.46	1.46	6.59	5.68	12.27
Endocrinology/Diabetology	0.38	0.38	0.32	0.35	0.27	0.27	0.22	0.32	1.43	1.08	2.51
Ophthalmology	0.49	0.19	0.35	0.35	0.27	0.22	0.16	0.24	1.38	0.89	2.27
Gynecology	0.16	0.27	0.22	0.24	0.38	0.24	0.27	0.30	0.89	1.19	2.08
Neurosurgery	0.24	0.11	0.14	0.08	0.08	0.03	0.03	0.14	0.57	0.28	0.85
Neurology	0.08	0.16	0.19	0.14	0.08	0.16	0.08	0.14	0.57	0.46	1.03
Radiology	0.22	0.05	0.22	0.05	0.19	0.05	0.05	0.16	0.54	0.45	0.99
Oncology/Haematology	0.00	0.03	0,00	0.00	0.00	0.00	0.00	0.00	0.03	0.00	0.03
Other	1.16	1.52	1.18	1.25	1.43	1.19	1.52	1.48	5.11	5.62	10.73

**Table 3:** Share of patients with TTR-HO with the most frequent outpatient diagnoses documented within the two years following TTR-HO-associated index hospitalization (n = 37).

	Top-10 diagnoses (ICD-10-GM)	Index	Post-observational period								
			Q1	Q2	Q3	Q4	Q5	Q6	Q7	Q8	Total
Oncological	Benign neoplasm: Pituitary gland (D35.2)	46%	51%	49%	46%	49%	46%	41%	43%	46%	59%
	Neoplasm of uncertain or unknown behavior: Pituitary gland (D44.3)	35%	32%	32%	32%	0%	24%	22%	27%	19%	54%
	Neoplasm of uncertain or unknown behavior: Brain, unspecified (D43.2)	22%	19%	0%	24%	22%	19%	14%	14%	0%	43%
	Neoplasm of uncertain or unknown behavior: Craniopharyngeal duct (D44.4)	22%	19%	27%	24%	27%	24%	24%	24%	22%	32%
Neuroendocrine	Hypopituitarism (E23.0)	27%	32%	41%	49%	54%	54%	46%	59%	46%	84%
	Diabetes insipidus/AVP-D (E23.2)	22%	41%	46%	59%	57%	62%	51%	49%	46%	81%
	Hypothyroidism, unspecified (E03.9)	0%	0%	22%	24%	16%	22%	19%	22%	22%	38%
Other	Obesity, unspecified: Degree or extent of obesity unspecified (E66.99)	27%	24%	30%	32%	22%	30%	27%	32%	24%	49%
	Essential hypertension, unspecified: Without indication of hypertensive crisis (I10.90)	24%	19%	27%	35%	27%	27%	22%	27%	22%	43%
	Visual field defects (H53.4)	24%	22%	0%	0%	0%	0%	0%	16%	0%	38%

Abbreviation: arginine vasopressin deficiency; AVP-D.

Similarly, management of central AVP-D began early, with 22% of patients consulting for this diagnosis in the index quarter (Table 3). This steadily increased to 57% of patients in the fourth follow-up quarter. During the two-year follow-up, about 80% of patients consulted for AVP-D, with no clear differences between age groups. Other common reasons for outpatient visits included 49% of patients consulting specifically for obesity, 43% of patients consulting for essential hypertension, and 38% of patients consulting for visual field defect. In each quarter, approximately 30% of patients consulted for obesity. During the follow-up period, the calculated average number of outpatient visits per quarter for hypopituitarism, AVP-D and obesity were 0.73, 0.79 and 0.58 visits, respectively.

### Prescription medication

During the 2-year follow-up period, the average number of active prescriptions was 5.5 per patient per quarter, with an average total of 22.1 unique medications prescribed over the follow-up period (Table 4). These included hormones to treat TTR-HO-related neuroendocrine disorders: secondary or tertiary forms of adrenal deficiency, hypothyroidism, hypogonadism, growth hormone deficiency, and hypopituitarism as a combination of all. Treatment for hypopituitarism was initiated rapidly following the index event, with 70% of patients receiving hydrocortisone during the first and 62% during the second follow-up quarter. Desmopressin therapy was also initiated rapidly, with 65% of patients receiving desmopressin during the first and 76% during the second follow-up quarter. DDDs per patient were high for neuroendocrine replacements, especially estradiol (512), dydrogesteron-estrogen (504), levothyroxine-sodium (473), and testosterone (356).

**Table 4:** Share of TTR-HO patients with the most frequent prescription medication within the first two years following TTR-HO-associated index hospitalization (n = 37).

Prescriptions drug		Index	Post observational period									
			Q1	Q2	Q3	Q4	Q5	Q6	Q7	Q8	Total	DDD/patient
Average number of prescriptions per patient		4.0	5.8	5.5	5.7	5.5	5.5	5.3	5.4	5.8	22.1	–
**Share of patients with specific medication**												
Neuroendocrine replacement	Desmopressin	38%	65%	76%	73%	62%	68%	70%	68%	65%	100%	339
	Hydrocortisone	59%	70%	62%	73%	68%	68%	73%	65%	65%	97%	344
	Levothyroxine-sodium	41%	46%	51%	76%	68%	68%	73%	68%	68%	86%	473
	Prednisone	8%	8%	0%	0%	0%	0%	0%	0%	0%	24%	26
	Somatropin	0%	0%	0%	0%	8%	8%	11%	0%	14%	16%	145
	Testosterone	0%	8%	16%	14%	22%	16%	16%	16%	16%	27%	356
	Dydrogesteron-Estrogen	0%	0%	0%	5%	0%	5%	0%	0%	0%	5%	504
	Progesterone	0%	0%	0%	0%	8%	0%	0%	5%	0%	8%	200
	Estradiol	0%	0%	0%	0%	0%	0%	5%	0%	5%	11%	512
	Estriol	0%	0%	0%	0%	0%	0%	0%	0%	5%	8%	186
Others	Ibuprofen	27%	5%	8%	19%	14%	16%	24%	16%	16%	68%	63
	Metamizole-Sodium	24%	19%	16%	14%	0%	11%	19%	11%	11%	65%	29
	Pantoprazole	32%	22%	32%	27%	22%	19%	22%	19%	22%	54%	742
	Cholecalciferol	5%	8%	11%	0%	5%	19%	16%	8%	11%	38%	164
	Diclofenac	8%	8%	0%	0%	14%	0%	0%	0%	0%	30%	106

Q: Quarter; Y: Year.

Note: Quarterly reporting based on Top 20 medications per quarter. Y2 reporting based on Top 20 medications overall in the two-year period. Total is reported cumulatively for all patients.

Most patients also received treatment for gastric reflux and other glucocorticoid-associated gastrointestinal complaints, either with pantoprazole (54%) or omeprazole (16%). Combined DDDs for these agents were high, at 991 DDDs/patient. Other common prescriptions included metoprolol and ramipril for hypertension (14% of patients each), and enoxaparin for coagulation disorder (19% of patients). In addition, 14% of patients received metformin for Type II diabetes. None of the patients living with TTR-HO received GLP-1 agonists within one year before index event or within the follow-up period.

**Fig. 1:**
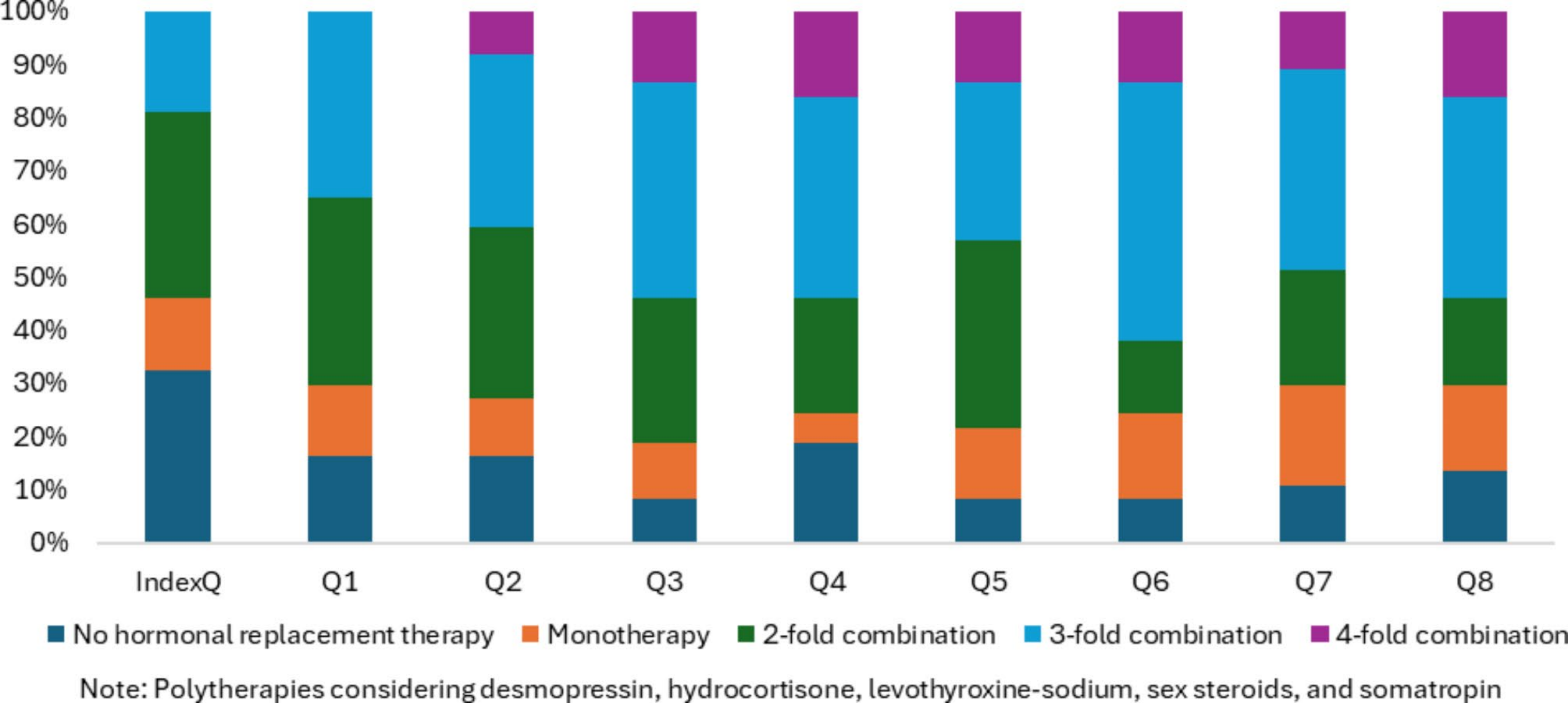
Percentage of patients with polytherapy for substitution of neuroendocrine deficits in patients with TTR-HO (per quarter following index event).

In the index event quarter, 68% of patients already had a prescription for a neuroendocrine replacement therapy and 19% of patients received a combination of three neuroendocrine therapies (the highest number in this quarter, Fig. 1). By the end of year 1, virtually all patients had a prescription to address neuroendocrine deficits and 89% were on a combination of three or more therapies at some point. In year 2, 68% of patients were on combination of three or more therapies in at least one quarter, while 14% of patients had one or fewer hormone replacement prescriptions during the year. In both years, about 25% of patients received a combination of 4 or more therapies for neuroendocrine deficits at some point in the year.

## Discussion

This detailed analysis of treatment pathways confirms the highly complex medical care and the need for close multidisciplinary collaboration when treating patients who develop TTR-HO following tumor/ treatment. In the first two years following index surgery/radiation therapy, patients return several times to the hospital. While several visits are related to oncological follow-up, there are also hospitalizations related to the management of disease- and/or treatment-related comorbidities which are attributable to TTR-HO and associated neuroendocrine deficits. Overall, treatment of neuroendocrine deficits is a critical part of the burden of TTR-HO. For example, of patients who develop TTR-HO, about 80% consult for AVP-D and about 84% consult for hypopituitarism during the study period. In contrast, of patients with tumors who do not develop obesity, only 2.3% had a documented diagnosis of AVP-D or hypopituitarism during the study period.

Furthermore, patients with TTR-HO are under close outpatient medical care, with an average of over thirty outpatient visits to general practitioners and various specialists over the follow-up period. In the German health care setting, general practitioners and endocrinologists have an important role in organizing access to specialists, ensuring that patients with TTR-HO understand their complex polytherapy regimen, and training patients to perform flexible dose adaptations if necessary. Specialists must be aware that the symptom(s) they manage are one of many and must dedicate time to review complex patient files and educate patients on the benefits of managing treatments and symptoms.

Likewise, patients and caregivers are often not prepared for this high burden of care, expecting tumor removal to be the conclusion of a stressful medical journey and not the start of a chronic one^26,27^. It is important for neurooncologists to prepare patients and caregivers prior to the tumor treatment procedure. A survey of eighty-two caregivers of craniopharyngioma survivors illustrates the unmet needs of survivors and their caregivers, associated with the survivor’s symptomatology impacting HRQOL^27^.

Treatment of TTR-HO and its co-morbidities is challenging, as several drugs used in managing neuroendocrine deficiencies and other symptoms in patients living with TTR-HO are known to induce weight gain^28^. These include hydrocortisone, used in 97% of patients, and prednisone, used in 24% of patients. Although physiologic replacement of hydrocortisone due to adrenal insufficiency would not promote weight gain, the long-term use of corticosteroids may contribute to the challenge of managing comorbidities and weight. Metoclopramide, which promotes fluid retention, was used in 19% of patients and cardiovascular drugs metoprolol and ramipril were used in 14% of patients each. While use of these therapies is dictated by the need to manage comorbidities, this can compound the weight gain and render attempts at losing weight ineffective.

In addition, there is a notable use of prescriptions for gastric reflux. It is well established that obesity contributes to the development or the exacerbation of gastrointestinal symptoms through a variety of mechanisms^29^. However, the risk of gastrointestinal complaints and gastric reflux is increased due to damage to the hypothalamus^4^, and by several hormonal replacement therapies such as hydrocortisone and estrogens^30^. Management of symptoms and prescriptions is a balancing act; treating physicians should take special care in teaching and training patients and caregivers to manage challenges in daily life of patients living with TTR-HO. Telematic solutions could make it easier for patients, especially those with mobility challenges, to receive consistent access to high-quality care.

TTR-HO is a distinct clinical entity that requires specific treatment and patient management. Unlike general obesity, which develops over time and can be managed by lifestyle changes, TTR-HO is related to fundamental changes in satiety and energy expenditure systems, leading to hyperphagia, reduced energy expenditure, and rapid weight gain^4,5^, and treatments for general obesity are largely ineffective for patients with TTR-HO^9^. Therefore, conventional recommendations around diet and exercise, which may be effective treatments for general obesity, are inadequate to address the underlying mechanisms in TTR-HO^9^. In a comparison of existing treatment modalities, Rose et al*.* find that a realistic goal for most patients is stable BMI rather than long-term BMI reduction^17^. Until now, no stand-alone pharmacological or bariatric approach has proven to be effective in long-term reduction of BMI for patients with TTR-HO^5^.

While currently available treatments show limited efficacy and considerable side effects, new treatment options for HO are under development. In particular, improvements in the understanding of neurological pathways enable the development of novel pharmacological approaches to regulating satiety and appetite in patients living with TTR-HO^31^. One example, setmelanotide, a melanocortin-4 receptor (MC4R) agonist, is currently in a phase 3 clinical trial for patients with lesional hypothalamic obesity, following robust phase-2-trial data showing a significant proportion of TTR-HO patients achieving ≥ 5% reduction in BMI^32^. It is important to note that there was no documented use of pharmacological weight loss therapies, such as GLP-1 agonists, in our study, likely because these drugs are not yet covered by the German statutory health system. However, it is possible that a patient could have self-paid for a prescription, which would be unobservable in statutory claims data.

This study stresses the high need for coordination of care of patients living with TTR-HO to ensure that each comorbidity is optimally managed within a holistic view of the individual patient. Careful, personalized monitoring can help identify early changes, such as the development of hyperphagia, in order to proactively manage weight gain^33^. Evidence that the severity of TTR-HO is highly dependent on the complexity of tumor treatment^34,35^, as well as evidence that morbidity and mortality are correlated with factors such as female sex, childhood onset, and tumor recurrence underline the importance of individualized monitoring of symptoms and comorbidities^36^.

One of this study’s primary strengths is the large data set, which allowed us to identify a relatively high number of patients living with TTR-HO in order to analyze patient pathways. However, the study design and methods face limitations. First, we are limited by the lack of a standardized definition of TTR-HO to identify the study population prior to the observational period. If, for example, there are long intervals between hospitalizations, a 1-year wash-in period could be too short, which could lead a prevalent case to be misclassified as an incident case. However, in the absence of a standardized definition of TTR-HO, the chosen approach represents the best possible approximation of a TTR-HO cohort for this setting. Second, as no clinical parameters such as BMI, laboratory test results, or information on health behavior are available in claims data, the analysis of weight gains is limited by the available diagnosis codes. Poor sensitivity of ICD-10 diagnosis data for administrative diagnosis of overweight/obesity has been reported in the literature^37^. However, it can be assumed that documentation might be more accurate because of the high patient relevance of rapid weight gain in TTR-HO. Third, the potential for upcoding must be considered for some validation criteria^38^. This effect should, however, be small due to our strict case definition (i.e., in the primary analysis, all validation criteria need to be documented within 4 quarters after index hospitalization). Fourth, self-selection bias may be present in the data. For example, patients with severe weight gain might be more likely to seek treatment (and be documented in claims data) than patients with fewer health problems after TTR-HO. Weight gain is a characteristic symptom of hypothalamic obesity as part of hypothalamic syndrome, which includes further morbidity. However, this effect is probably small for the validation criteria applied in this study, as symptoms are typically severe and diagnosed by the physician, which makes the classification less likely to be influenced by self-selection.

Future research should seek to compare treatment pathways between patients with tumors who develop TTR-HO and those who do not; this would provide valuable insight on the medical burden due exclusively to obesity and metabolic comorbidities. In addition, future research should seek to identify patterns in the development of TTR-HO following surgery versus radiation.

## Conclusion

Although it is difficult to disentangle the medical burden of TTR-HO from the general burden of tumor survivorship, we provide important detail on the pathways of patients who develop TTR-HO following tumor treatment. TTR-HO is a rare and debilitating disease with an impact on patients’ health and quality of life. Treatment of TTR-HO is challenging, requiring frequent contact with multiple specialists, a complex regimen of medications, and sometimes hospitalization. The burden of care is high for patients, caregivers, and treatment teams, especially as treatment of comorbidities can contribute to the ongoing challenge of weight management. The major tasks in treatment of TTR-HO are balancing complex neuroendocrine replacement therapies and the interaction of symptoms, therapies, and co-morbidities. Centralized treatment and close monitoring could make treatment of TTR-HO more effective and manageable.

## Electronic supplementary material

Below is the link to the electronic supplementary material.

Supplementary Material 1

## Data Availability

The data that support the findings of this study are available from GWQ ServicePlus AG but restrictions apply to the availability of these data, which were used under license for the current study, and so are not publicly available. Data are however available from the authors upon reasonable request and with permission of GWQ ServicePlus AG. To request the data from this study, please contact the corresponding author at kylie.braegelmann@vandage.de.
