# Supplementary material for: Treatment of patients with tumor/treatment-related hypothalamic obesity in the first two years following surgical treatment or radiotherapy
Source: Sci Rep. 2025 Jan 16;15:2118. doi: 10.1038/s41598-025-85262-1 (PMC11736136; doi:10.1038/s41598-025-85262-1)
Supplement: Supplementary file 1 — Supplementary Material 1 [file 41598_2025_85262_MOESM1_ESM.pdf]

# Supplementary Appendix

## Contents

**Table S1. Top-10 secondary diagnoses of follow-up hospitalizations within 2 years after index hospitalization** .......... 2

**Table S2. Mean number of hospitalizations per HO patient by age groups** .......... 3

**Table S3. Top-10 outpatient diagnoses within the two years following HO-associated index event (patient group < 20 years)** .......... 4

**Table S4. Top-10 outpatient diagnoses within the two years following HO-associated index event (patient group 20-59 years)** .......... 5

**Table S5. Top-10 outpatient diagnoses within the two years following HO-associated index event (patient group 60+ years)** .......... 6

**Table S1. Top-10 secondary diagnoses of follow-up hospitalizations within 2 years after index hospitalization**

| ICD-10-GM diagnosis | Description | Share of hospital-ized HO-patients with diagnosis |
|---|---|---|
| E23.2 | Diabetes insipidus/arginine vasopressin deficiency (AVP-D) | 35% |
| E23.0 | Hypopituitarism | 21% |
| E87.6 | Hypokalemia | 18% |
| I10.00 | Benign essential hypertension: without indication of hypertensive crisis | 16% |
| R51 | Headache | 15% |
| E03.9 | Hypothyroidism, unspecified | 12% |
| H53.4 | Visual field defects | 12% |
| R11 | Nausea and vomiting | 12% |
| E03.8 | Other specified hypothyroidism | 10% |
| E87.0 | Hyperosmolality and hypernatremia | 9% |

HO, hypothalamic obesity; ICD-10-GM, International classification of diseases, Tenth revision, German Modification.

**Table S2. Mean number of hospitalizations per HO patient by age group**

| Age group | Hospitalization | Index quarter | Q1 | Q2 | Q3 | Q4 | Q5 | Q6 | Q7 | Q8 | Y1 | Y2 |
|---|---|---|---|---|---|---|---|---|---|---|---|---|
| **< 20** | HO-related | 1.50 | 0.67 | 0.83 | 0.50 | 0.83 | 0.17 | 0.50 | 0.33 | 0.17 | 2.83 | 1.17 |
| | Non-HO-related | 0.17 | 0.17 | 0.00 | 0.00 | 0.17 | 0.00 | 0.17 | 0.00 | 0.17 | 0.33 | 0.33 |
| **20 – 59** | HO-related | 1.23 | 0.23 | 0.15 | 0.12 | 0.00 | 0.15 | 0.08 | 0.00 | 0.00 | 0.50 | 0.23 |
| | Non-HO-related | 0.65 | 0.77 | 0.54 | 0.31 | 0.27 | 0.15 | 0.15 | 0.15 | 0.31 | 1.88 | 0.77 |
| **60+** | HO-related | 1.60 | 0.20 | 0.20 | 0.60 | 0.00 | 0.00 | 0.00 | 0.00 | 0.00 | 1.00 | 0.00 |
| | Non-HO-related | 1.00 | 0.40 | 0.60 | 0.60 | 0.00 | 0.40 | 0.60 | 0.20 | 0.20 | 1.60 | 1.40 |

HO, hypothalamic obesity

**Table S3. Top-10 outpatient diagnoses within the two years following HO-associated index event (patient group < 20 years)**

| ICD-10-GM Diagnosis | Description | Share of patients | Physician contacts per patient (per quarter) |
|---|---|---|---|
| D44.4 | Neoplasm of uncertain or unknown behavior: Cra-niopharyngeal duct | 83% | 8.0 |
| D43.2 | Neoplasm of uncertain or unknown behavior: Brain, unspecified | 67% | 6.5 |
| E23.0 | Hypopituitarism | 100% | 4.3 |
| E23.2 | Diabetes insipidu/arginine vasopressin deficiency (AVP-D) | 83% | 3.0 |
| F43.2 | Adaptation disorders | 50% | 4.3 |
| C71.9 | Malignant neoplasm: brain, unspecified | 17% | 12.0 |
| E66.99 | Obesity, unspecified: Degree or extent of obesity unspecified | 33% | 6.0 |
| H53.4 | Visual field defects | 33% | 6.0 |
| D35.2 | Benign neoplasm: Pituitary gland | 17% | 11.0 |
| H53.9 | Visual impairment, unspecified | 33% | 5.0 |

HO, hypothalamic obesity.

**Table S4. Top-10 outpatient diagnoses within the two years following HO-associated index event (patient group 20-59 years)**

| ICD-10-GM Diagnosis | Description | Share of patients | Physician contacts per patient (per quarter) |
|---|---|---|---|
| D35.2 | Benign neoplasm: Pituitary gland | 73% | 14.3 |
| E23.2 | Diabetes insipidus/ arginine vasopressin deficiency (AVP-D) | 81% | 8.7 |
| E23.0 | Hypopituitarism | 85% | 7.4 |
| D44.3 | Neoplasm of uncertain or unknown behavior: Pituitary gland | 65% | 5.8 |
| E03.9 | Hypothyroidism, unspecified | 38% | 7.0 |
| D44.4 | Neoplasm of uncertain or unknown behavior: Craniopharyngeal duct | 19% | 13.6 |
| E66.99 | Obesity, unspecified: Degree or extent of obesity unspecified | 50% | 5.0 |
| Z01.7 | Laboratory examination | 27% | 7.9 |
| I10.90 | Essential hypertension, unspecified: Without indication of hypertensive crisis | 38% | 5.2 |
| F32.9 | Depressive episode, unspecified | 31% | 6.3 |

HO, hypothalamic obesity.

**Table S5. Top-10 outpatient diagnoses within the two years following HO-associated index event (patient group 60+ years)**

| ICD-10-GM Diagnosis | Description | Share of patients | Physician contacts per patient (per quarter) |
|---|---|---|---|
| I10.90 | Essential hypertension, unspecified: Without indication of hypertensive crisis | 100% | 10.0 |
| D35.2 | Benign neoplasm: Pituitary gland | 40% | 15.0 |
| D44.4 | Neoplasm of uncertain or unknown behavior: Craniopharyngeal duct | 40% | 13.0 |
| H26.9 | Cataract, unspecified | 60% | 8.0 |
| D43.2 | Neoplasm of uncertain or unknown behavior: Brain, unspecified | 40% | 9.5 |
| M54.5 | Low back pain | 60% | 6.3 |
| E79.0 | Hyperuricemia without signs of inflammatory arthritis or tophic gout | 40% | 9.0 |
| E66.99 | Obesity, unspecified: Degree or extent of obesity unspecified | 60% | 5.7 |
| E23.2 | Diabetes insipidus/ arginine vasopressin deficiency (AVP-D) | 80% | 4.0 |
| E23.0 | Hypopituitarism | 60% | 5.0 |

HO, hypothalamic obesity.
